# Pre-stimulus EEG Microstates Correlate With Anticipatory Alpha Desynchronization

**DOI:** 10.3389/fnhum.2020.00182

**Published:** 2020-05-27

**Authors:** Sara Spadone, Pierpaolo Croce, Filippo Zappasodi, Paolo Capotosto

**Affiliations:** ^1^Department of Neuroscience Imaging and Clinical Sciences, University “G. d’Annunzio”, Chieti, Italy; ^2^Institute for Advanced Biomedical Technologies University “G. d’Annunzio”, Chieti, Italy

**Keywords:** anticipatory period, EEG, alpha rhythms, pre-stimulus microstates, semantic memory

## Abstract

In the last decades, several electrophysiological markers have been investigated to better understand how humans precede a signaled event. Among others, the pre-stimulus microstates’ topography, representing the whole brain activity, has been proposed as a promising index of the anticipatory period in several cognitive tasks. However, to date, a clear relationship between the metrics of the pre-stimulus microstates [i.e., the global explained variance (GEV) and the frequency of occurrence (FOO)] and well-known electroencephalography marker of the anticipation (i.e., the alpha power reduction) has not been investigated. Here, after extracting the microstates during the expectancy of the semantic memory task, we investigate the correlations between the microstate features and the anticipatory alpha (8–12 Hz) power reduction (i.e., the event-related de-synchronization of the alpha rhythms; ERD) that is widely interpreted as a functional correlate of brain activation. We report a positive correlation between the occurrence of the dominant, but not non-dominant, microstate and both the mean amplitude of high-alpha ERD and the magnitude of the alpha ERD peak so that the stronger the decrease (percentage) in the alpha power, the higher the FOO of the dominant microstate. Moreover, we find a positive correlation between the occurrence of the dominant microstate and the latency of the alpha ERD peak, suggesting that subjects with higher FOO present the stronger alpha ERD closely to the target. These correlations are not significant between the GEV and all anticipatory alpha ERD indices. Our results suggest that only the occurrence of the dominant, but not non-dominant, microstate should be considered as a useful electrophysiological correlate of the cortical activation.

## Introduction

Electroencephalography (EEG) records the human brain electric activity with a high temporal and a reasonably good spatial resolution. Due to its features, this is a popular neuroimaging modality for understanding how humans precede and prepare for a signaled event, providing several neural correlates in both time and frequency domains. In this regard, the alpha (8–12 Hz) power reduction (event-related de-synchronization, ERD) is a typical marker of the neural mechanisms that contribute to the development of temporal expectations, and it is widely observed in the period that precedes a target in a variety of cognitive tasks (Min et al., 2008; Capotosto et al., 2009, 2015; Babiloni et al., 2014). Such alpha power reduction is mostly observed in the upper alpha sub-band (∼10–12 Hz), which is the sub-band thought to reflect the task-related oscillation of specific neural systems for the elaboration of signaled information, whereas the lower alpha sub-band (∼8–10 Hz) is thought to reflect general arousal and vigilance (Klimesch et al., 1998b).

Importantly, in paradigms using a fixed temporal period between a warning and an event stimulus, the amplitude of alpha ERD increases rhythmically and peaks just before the target (Rohenkohl and Nobre, 2011). Among others, a clear alpha ERD has been reported during the preparatory period of a semantic memory task (reviewed in Klimesch et al., 2010, in which a warning signal preceded simple semantic judgments (e.g., living vs. non-living) and the alpha power became suppressed during the preparatory period as the subject anticipated the beginning of the successive trial. Accordingly, the time modulation of the anticipatory alpha power was also observed during the execution of a semantic memory task (Spadone et al., 2017). Although it is widely adopted to investigate the period that precedes an expected event, the alpha ERD measure, as other common EEG correlates of anticipation in the time domain (i.e., contingent negative variation, CNV), has some limitations, e.g., its regional specificity. Moreover, this measure requires evaluation of an epoch lasting one or more seconds. On the contrary, the whole brain is continuously in a different state (Kondakor et al., 1997).

In recent years, similarly to the reduction of the alpha power, another EEG correlate has been proposed for the characterization of the brain activity in the period preceding an expected event, i.e., the dominant pre-stimulus microstate obtained by clustering one map from each trial in the 50 ms before the event (Croce et al., 2018b). EEG microstate analysis considers the EEG signal from all electrodes to create a global representation of a functional state, thus providing an overall view of the human brain activity in the sub-second range. The topography of the EEG microstates in a period that precedes a signaled target has been investigated in a series of cognitive tasks (Britz et al., 2008, 2011, 2014), and it is considered a proper index to assess the information processing currently performed by the brain. Albeit the increasing interest in the study of the microstates both at rest and during the task execution, their brain functional dynamics, also compared to other EEG markers, are still debated.

Recently, Milz et al. (2017) explored whether the resting state EEG microstate’s topographies are driven by specific brain rhythm activity. They linked the emergence of the four typical microstate topographies at rest (A, B, C, and D) with the intra-cortical alpha oscillations, suggesting an association between EEG frequency band-wise differences and the EEG microstates. To date, a similar relationship is not still investigated during the task execution although both the anticipatory alpha de-synchronization and the pre-stimulus microstates provide important information on the neural mechanisms relative to the anticipatory processes. To this aim, here, we extract the pre-stimulus microstates during the expectancy of a semantic memory task, and we investigate the correlations between the microstate features (i.e., the global explained variance, GEV, and the frequency of occurrence, FOO) and the anticipatory alpha ERD indices describing both the whole anticipatory period (mean amplitude) and the dynamics of alpha band modulation (latency and magnitude of the alpha ERD peak).

## Materials and Methods

### Subjects and Stimuli

Eighteen right-handed (Oldfield, 1971) volunteers (mean age ± SD = 28.5 ± 4.9 years old; 11 females), same sample as in Capotosto et al. (2017), with no previous psychiatric or neurological history participated in the experiment. All experiments were conducted with the understanding and written informed consent of each participant, according to the Code of Ethics of the World Medical Association and the standards established by the University of Chieti Institutional Review Board and Ethics Committee. The experimental protocol was approved by the Institutional Review Board and Ethics Committee of the University of Chieti (prot. 1123/2014). Of note, whereas our referenced study (Capotosto et al., 2017) included experimental runs with EEG recording combined with transcranial magnetic stimulation (TMS), here we only refer to the experimental condition in which the interference was ineffective (i.e., Sham), so that the present EEG signals are not contaminated by the magnetic stimulation.

**FIGURE 1:**
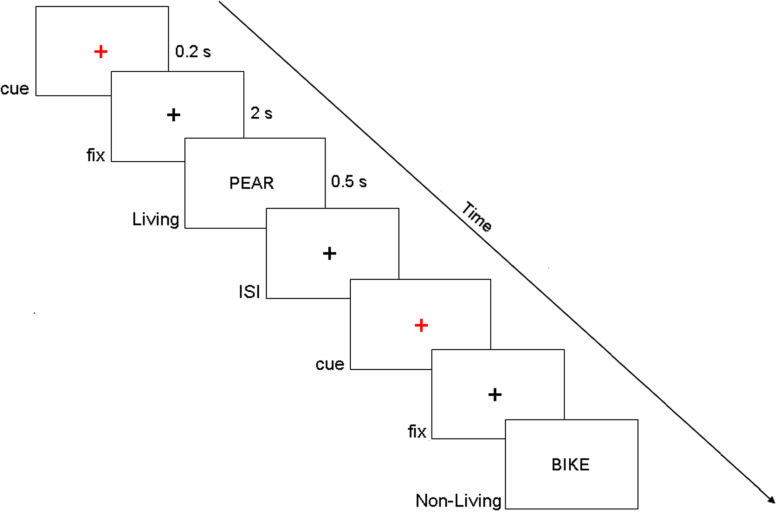
Example of the display sequence in the semantic memory task. Of note, in the present experimental paradigm, we investigated the period that precedes the target onset (i.e., fix period in this figure).

The participants were seated on a comfortable reclining armchair and kept their hands on the response box (Cedrus RB-830). Stimuli were generated using E-Prime software v2.0 (Psychological Software Tools, Pittsburgh, PA, United States) and were presented on an LCD screen placed at a distance of about 80 cm. They represented four-letter actual Italian nouns (words were written in uppercase), drawn from a linguistic database [Corpus e Lessico di Frequenza dell’Italiano Scritto (CoLFIS), Bertinetto et al., 2005]. Subjects were instructed to maintain fixation on a central black cross (subtending 0.2° of visual angle), displayed on a white background at the center of the screen. Every 4 ± 0.5 s, a cue stimulus (a small red cross) was presented for 200 ms. After 2 s, a word was presented for 500 ms at the center of the screen and denoted a living (50%) or a non-living (50%) entity. Participants, within the present semantic decision task, were instructed to make a living/non-living judgment by pressing a corresponding button of the response box with their left/right index finger (Figure 1) and to respond as quickly and as accurately as possible. Of note, living/non-living subcategories are not considered separately in the following analyses, and they include plants (e.g., vegetables, fruits, flowers), animals (e.g., birds, mammals, insects), and body parts for the living category and buildings, vehicles, apparel, music instruments, and tools for the non-living category. Fifty trials per subject were collected, but only correctly answered trials were kept for the following analyses (83.7 ± 1.7%).

### Electroencephalography Recordings

Electroencephalography data were recorded (BrainAmp; bandpass, 0.05–100 Hz, sampling rate, 256 Hz; AC couple mode recording; notch filter not applied during the recording) from 32 EEG electrodes placed according to an augmented 10–20 system and mounted on an elastic cap. Electrode impedance was set below 5 kOhm. The reference electrode was placed between Fz and Cz, whereas the ground electrode was positioned below Oz. Two electro-oculographic channels (EOG, i.e., one horizontal and one vertical, 0.05–100 Hz bandpass) were used to monitor eye movements and blinking. The EEG was recorded continuously and then was off-line segmented in epochs lasting from −1 s before and +3.5 s after cue onset. For the EEG analyses, a second-order filter was applied, 1–40 Hz bandpass backward–forward. EEG trials contaminated by eye movements, blinking, or other involuntary movements (e.g., mouth, head, trunk, or arm) were off-line rejected by visual inspection. EEG single trials were re-referenced by the common average reference, which includes the averaging of amplitude values at all electrodes and the subtraction of the mean value from the amplitude values at each single electrode. Notably, for all subjects, no bad channels were present. Following artifact removal, an average number of 40 (±2.5) trials per subject were available for the EEG analysis.

### Electroencephalography Analysis

First, we determined the peak of individual alpha frequency (IAF) for each subject from −1 to 0 s before the cue onset (rest period). The IAF peak was defined as the maximum power density peak between 6 and 14 Hz considering the average of the EEG power density across all electrodes. This frequency landmark was well described by Dr. Wolfgang Klimesch and his workgroup (Klimesch et al., 1998a; Klimesch, 1999). In a few words, the IAF is defined as the frequency associated to the strongest EEG power at the extended alpha range and is the anchor point for distinguishing a lower from an upper alpha band. With respect to the IAF, these frequency bands are defined as follows: (i) low alpha, IAF –2 Hz to IAF and (ii) high alpha, IAF to IAF +2 Hz. For example, if a subject has the IAF at 10 Hz, we averaged the frequencies as follows: (i) for low alpha, averaging across 8, 9, and 10 Hz; for high alpha, averaging across 10, 11, and 12 Hz; and for the whole alpha band, across 8–12 Hz. Of note, the mean of the low alpha band across subjects was 9.3 ± 0.8 Hz, whereas the mean of the high alpha band across subjects was 11.3 ± 0.8 Hz. To this aim, the power spectrum was estimated by means of a fast Fourier transform (FFT) approach (the Welch method with a Hanning windowing function). An EEG period of 1 s was used as input for FFT. Two different power spectra were computed: one in the “rest” period, considering EEG windows from −1 to 0 s before the cue onset, and one in the “event” period, i.e., from −1 to 0 s before target onset. The event-related de-synchronization/synchronization (ERD/ERS) of alpha EEG oscillations was obtained using

ERD%=(E-R)/R×100

where E indicates the power density at the event (lasting 1 s) and R the power density at the rest (lasting 1 s). Hence, negative ERD values indicated a reduction in alpha power in the event compared to the rest period. Notably, this stationary analysis was performed on the regional average of five parietal-occipital electrodes (i.e., P7, P8, O1, O2, and Oz) that were selected as alpha activity is most consistently localized in the parietal-occipital cortex (Vanni et al., 1997). The mean obtained with such analysis was correlated with the metrics of the dominant and non-dominant EEG microstates.

Next, a time-frequency analysis was carried out to investigate the relationship between the microstates’ metrics and the dynamics of the alpha ERD in terms of peak latency and maximal power decrease. To this aim, time-frequency representations were obtained by means of the Morlet wavelets for each individual trial and then averaged across trials (Tallon-Baudry et al., 1997; Jensen et al., 2002). The frequency and temporal bins were 1 Hz and 4 ms, respectively. We set the wavelet width to seven cycles as a balance between temporal and frequency resolution. To avoid edge effects, EEG trials were segmented from −1.2 to 0.2 s with respect to the target onset, and after wavelet transform, 0.2 s at both sides were discarded, obtaining a period from −1 to 0 s before the target onset. For all subjects, we computed the ERD/ERS of alpha EEG oscillations as a function of frequency and time as instantaneous percentage power variations in the pre-target period with respect to mean power in the pre-cue period. Notably, in the low-alpha sub-band, the ERD peak was not clearly detectable in many subjects, and several subjects presented a tiny ERS peak. Accordingly, we observed that the mean value of the responsive frequency of the whole individual alpha ERD (10.9 Hz) was higher than the mean of IAF across subjects (10.3 Hz). Thus, we calculated the latency and the magnitude of the ERD peak on the whole individual alpha band (from IAF −2 Hz to IAF +2 Hz) averaging across the five frequency positions (i.e., IAF −2 Hz, IAF −1 Hz, IAF, IAF +1 Hz, IAF +2 Hz) the percentage power variations. Individual latencies and relative magnitude of ERD peak were automatically measured at the global minimum of the corresponding time course with reference to the target onset time. Of note, both preprocessing and data analyses were performed by homemade Matlab programs. Importantly, such time-frequency analysis was performed on the same regional average of five parietal-occipital electrodes (i.e., P7, P8, O1, O2, and Oz) previously used for the stationary analysis.

### Microstates Analysis

Microstate pre-stimulus analysis aims to find the most representative maps in a time interval before the stimulus. To obtain these maps, we followed the procedure described by Britz et al. (2014) and also applied in Croce et al. (2018a, b) by using EEGlab microstates toolbox (Delorme and Makeig, 2004). Standard deviation of EEG signals across electrodes is known as global field power (GFP). Considering that the maxima of GFP represent periods of highest topographic stability (Murray et al., 2008), for each subject and for each trial, we extracted the map corresponding to the maximum of the GFP in the period that precedes the target (i.e., 50 ms before the target). Specifically, we extracted one map for each trial for each subject. Such maps were jointly submitted to a modified version of the k-means algorithm proposed by Pascual-Marqui et al. (1995) varying the number of the cluster between two and 18. Notably, the cluster algorithm was tuned also to take into account the maps’ polarity. The optimal number of clusters (on average, *N* = 10) was chosen through the Krzanowski–Lai (KL) criterion. Considering the number of trials per subject (*N* ∼ 40), an average of *N* = 4 maps per cluster should be expected. It can be argued that this proportion may be too low, and in some cases, one or more clusters may contain only one template, and then it should be considered as an outlier. Nevertheless, using a different/lower number of clusters, possible outliers are forced to be wrongly assigned to other centroids, thus leading to a lower value of the KL criterion. Furthermore, the present proportion is consistent with the referenced study (Britz et al., 2008). Because the KL is a relative measure of dispersion (tending to zero when the clustering improves), the KL peaks should represent the optimal number of clusters. However, a KL peak is often observed when the whole set of data is separated into two clusters. Thus, as suggested in Murray et al. (2008), we chose the second peak of KL as representative of the optimal number of clusters.

Next, we statistically determined the most persistent topography, comparing both the GEV and the FOO of maps obtained from the clustering procedure. Separately for each subject, the maps previously extracted (subject-wise pre-stimulus templates, on average, *N* = 10) were assigned to each time frame map in the 50 ms prior to the cue stimulus based on the best spatial correlation, thus allowing the computation of the GEV and the FOO. The GEV is defined as

$$G\,E\,V=\frac{\sum_{t=1}^{t\,m\,a\,x}{\left(G\,F\,P_{u}\,\left(t\right)\,C_{u,T}\right)}^{2}}{\sum_{t=1}^{t\,m\,a\,x}G\,F\,P_{u}^{2}\,\left(t\right)}$$

where *C*_*u, T*_ is the spatial correlation between map *u* and template *T* and GFP is the global field power at the time *t*.

The FOO is defined as the number of occurrences of a certain microstate in a fixed time interval. In this step of analysis, the number of obtained maps is different for each subject.

**FIGURE 2:**
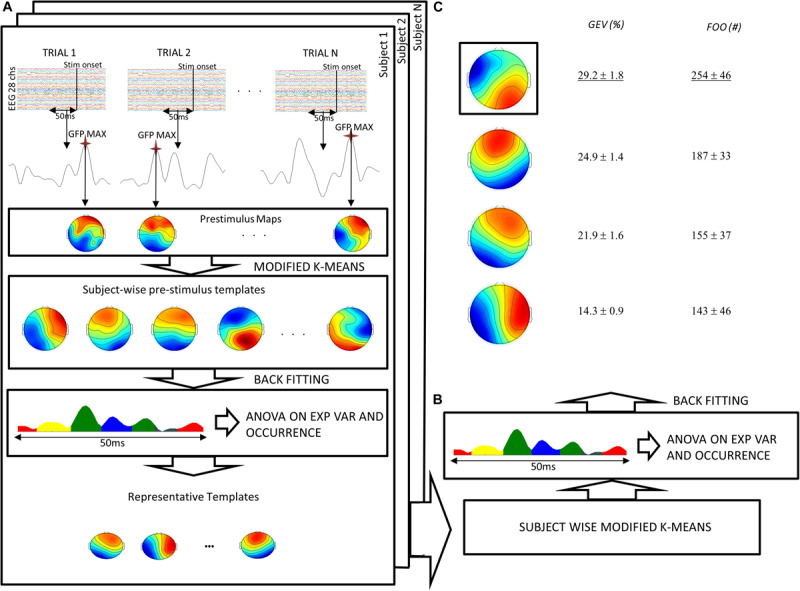
Flow chart of the pre-stimulus microstate analysis procedure. **(A)** For each subject and for each trial, the maximum of the GFP in the 50 ms preceding the target stimulus was submitted to a modified version of the k-means algorithm. The maps resulting from this procedure were then statistically tested (ANOVA) to obtain a certain number of representative templates for each subject. **(B)** Such maps were submitted to another k-means obtaining four templates. **(C)** These templates were fitted to the original data to obtain global explained variance (GEV) and frequency of occurrence (FOO). ANOVA and *post hoc* tests showed significant difference of GEV and FOO across templates. Finally, the template with maximal GEV and FOO was chosen as the “dominant template.” Notably, both GEV and FOO values have been averaged across subjects after summing all trials for each subject.

To differentiate the most representative maps, for each subject, an analysis of variance (ANOVA) followed by paired *t* test was performed on both GEV and FOO with the maps previously extracted as a within-subject factor. With this procedure, we extracted the maps that are more representative of the pre-stimulus time interval for each subject (choosing the templates with maximum GEV and FOO) (Figure 2A, last row). Next, to obtain only one representative map, we grouped all subject-wise maps obtained in the previous step and performed another k-means clustering (Pascual-Marqui et al., 1995) searching for four templates in order to take into account the inter-subject variability coming from the previous clustering (Britz et al., 2011; Figure 2B). The obtained templates (four in this case) were back-fitted to the original data to compute GEV and FOO. Importantly, the centroids of this second clustering were different with respect to centroids computed individually in the first clustering, and in this second step, the observations were forced to be assigned to one of the four group-wise templates. Thus, at this step, each subject presents a GEV and FOO (≥0) for each group-wise template. Within these four templates, dominant (DT, i.e., the template with maximum GEV and FOO) and non-dominant templates were statistically detected. Again, ANOVAs (within-subject factor the four templates previously obtained) followed by *post hoc* paired *t* tests were performed to assess that the GEV and FOO of these templates were different (Figure 2C). Nevertheless, we considered only three microstates (DT, C2, and C3) for the following considerations. Within the 50-ms pre-stimulus interval, *N* = 12 time frames were extracted. Thus, considering that the KL criterion detected *N* = 4 templates, it should be expected that, on average, each trial contained three time frames per template. In the case that one or more subjects did not present at least one trial with three or more time frames, the corresponding template was considered negligible. In particular, in the present study, the microstate C4 was excluded because, in several subjects, it was present less than three time frames in the whole set of trials, and in *N* = 2 subjects it was often missing. On the contrary, the dominant microstate as well as C2 and C3 occurred at least three times in one or more trials in each subject. Importantly, in the present data set, the maximum GEV and FOO always occurred for the same microstate.

First, within each data set (i.e., the four alpha ERD indices and the two metrics of the three microstates), we tested for outliers with Dixon’s test. Then, we investigated the link between the different EEG markers, computing several correlations (Spearman rank, *p* < 0.05) between the alpha ERD indices (i.e., mean amplitude, peak magnitude, and peak latency) and the dominant and non-dominant microstates’ features (i.e., the GEV and the FOO). Of note, compared to Pearson’s correlation coefficient, Spearman’s rank correlation provides a more robust measurement, especially using small samples, such as *N* = 18 (Schwarzkopf et al., 2012), and it is less sensitive to strong outliers. Then, we applied FDR correction considering the 24 correlations performed. Moreover, to verify whether the above correlations are specific for the dominant microstate, we statistically compared (i.e., Olkin’s test; Marshall and Olkin, 1967) the power of correlations of dominant and non-dominant microstates. Specifically, we tested the equality of the two correlation coefficients (dominant vs. non-dominant C2 and dominant vs. non-dominant C3 microstates) obtained from the same sample, considering that the two correlations share one variable (the alpha ERD indices). Finally, the features showing significant correlation (i.e., the occurrence of dominant microstate vs. the mean amplitude of high-alpha ERD, magnitude, and latency of the ERD peak) were linked to behavioral effects. Specifically, to assess the functional significance of these electrophysiological correlates, we estimated the Spearman rank correlation (*p* < 0.05) between EEG properties and behavior performance (i.e., reaction time and accuracy).

## Results

**FIGURE 3:**
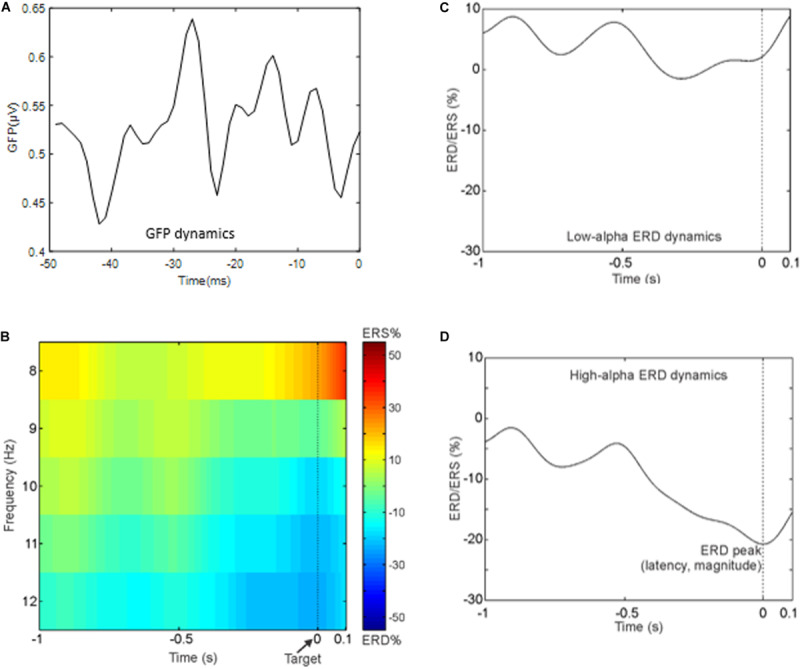
**(A)** Time course of the global field power in the 50 ms preceding the target onset. **(B)** Time–frequency representation averaged across subjects of the ERD in the whole alpha band in the period that precedes the target. Notably, negative values indicate a reduction in alpha power in the pre-stimulus interval (i.e., event period) compared to rest period. **(C)** Grand average ERD waveform in the low-alpha band. **(D)** Grand average ERD waveform in the high-alpha band.

Dominant and non-dominant pre-stimulus microstates were extracted in the period that precedes the semantic memory task (see section “Materials and Methods” and Figure 2 for a complete description). In this period, we observed the maximum of the GFP at about 24 ± 1.4 ms (median latency of 24.5 ms, minimum–maximum: 19 ms, 29 ms). Figure 3A shows the time course of the GFP in the period of interest (i.e., 50 ms before the target onset). Next, we identified the subject-wise pre-stimulus maps. The KL criterion provided an average number of optimal templates of 10.5 ± 2 per subject, explaining 78.9 ± 2.85 of the global variance. To compute both individual GEV and FOO, all subject-wise pre-stimulus maps were fitted to the original data. Specifically, we statistically determined the most persistent topography comparing both GEV and FOO obtained from the clustering procedure. The average number across subjects of these maps was 2 ± 1 with on average *p* value < 0.02. Such maps were then submitted to another k-means clustering algorithm searching a fixed number of clusters (i.e., *N* = 4). The GEV, considering the four templates, was 90.4%. Figure 2C shows the topography of the four maps (i.e., DT underlined and the three non-dominant microstates: C2, C3, and C4) obtained along with the values of GEV and FOO. Occurrence values considered for the correlation analysis are the sum of the occurrences of each trial. Such individual sums were then averaged across subjects. Notably, for the following correlations, we considered only three microstates (DT, C2, and C3) as explained in the Methods section.

For illustrative purpose, Figure 3B shows the group-averaged time-frequency pattern for the alpha band, and Figures 3C,D show the grand average ERD waveform in the low- and high-alpha sub-bands, respectively. It can be noted that the power decrement in the alpha band preceding the presentation of the target is as expected in a paradigm using a fixed cue–target interval. Moreover, the alpha power does not continue to decrease after the target onset. In particular, in what follows, we report the mean values and standard errors of the ERD indices: mean amplitude of the low-alpha ERD – 4.9 ± 5.9%; mean amplitude of the high-alpha ERD – 16.5 ± 4.9%; the magnitude of the alpha ERD peak – 21.3 ± 6.5%; the latency of the alpha ERD peak – 0.25 ± 0.06 s (i.e., the target onset is presented at 0 time).

The correlations between the metrics of the pre-stimulus microstates and the features of alpha power reduction (i.e., mean amplitude of the low- and high-alpha ERD and latency and magnitude of the alpha ERD peak) were investigated with Spearman’s rank, which is less sensitive to strong outliers compared to other correlation analyses (i.e., Pearson’s correlation coefficient). Before evaluating such a relationship, we computed Dixon’s test to check for possible outliers within each data set (i.e., the four alpha ERD indices and the two metrics of the three microstates). Results showed no significant outliers (*p* > 0.1) in all data sets except for one (tendency) outlier in the high-alpha mean amplitude (*p* = 0.051). For sake of clarity, the ERD amplitude values have been ranked from the minimum to the maximum decrease (percentage) in the alpha power (i.e., from 0 to 100% of power decrease).

**FIGURE 4:**
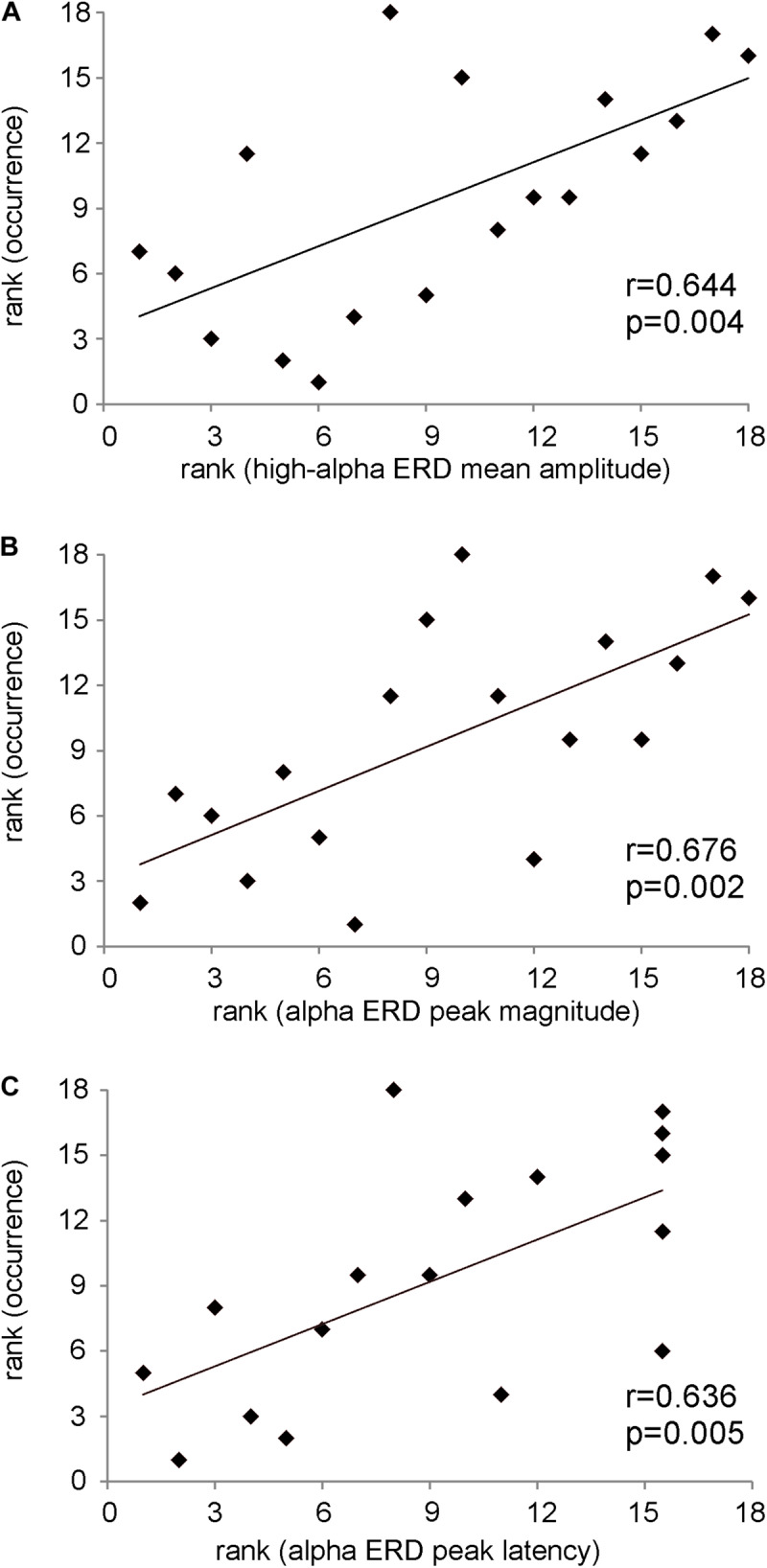
**(A)** Scatterplot showing the positive Spearman’s rank correlation between the mean amplitude of high-alpha ERD and the occurrence of the dominant microstate. **(B)** Scatterplot showing the positive Spearman’s rank correlation between the magnitude of the alpha ERD peak and the occurrence of the dominant microstate. **(C)** Scatterplot showing the positive Spearman’s rank correlation between the alpha ERD peak latency and the occurrence of the dominant microstate. On note, these *p* values are statistically significant after FDR correction.

In Figure 4A, the significant positive Spearman rank correlation between the mean amplitude of high-alpha ERD and the occurrence of the dominant microstate considered as the sum of the occurrences across trials (*r* = 0.644, *p* = 0.004) is reported. Notably, such correlation remains significant (*p* < 0.01) with a group of *N* = 17 subjects after removing the “partial” outlier. Interestingly, this correlation is lost when we considered the low-alpha ERD (*p* = 0.6). Consistently, Figure 4B shows the significant positive Spearman rank correlation between the magnitude of the alpha ERD peak and the occurrence of the dominant microstate (*r* = 0.68, *p* = 0.002). The above two significant correlations suggest that the stronger the decrease (percentage) in the alpha power ERD (both mean amplitude and peak magnitude of alpha ERD), the higher the FOO of the dominant microstate. Furthermore, we observed a significant positive Spearman rank correlation between the alpha ERD peak latency and the occurrence of the dominant microstate (*r* = 0.636, *p* = 0.005), showing that subjects with higher FOO tend to peak more in proximity to the target (Figure 4C). Conversely, there are no significant correlations between the alpha ERD indices and the GEV. Moreover, for both the FOO and the GEV of the non-dominant microstates, we observed no statistically significant correlation with any of the anticipatory alpha ERD indices (i.e., mean and peak amplitude and latency). Notably, the *p* values of all correlations between alpha ERD indices and microstate metrics are reported in Table 1.

**TABLE 1:** *P* and *r* values of Spearman-rank correlations between alpha ERD indices and microstate metrics.

	***DT-occ***	***C2-occ***	***C3-occ***	***DT-gev***	***C2-gev***	***C3-gev***
***p value***						
*Low-alpha*	*0.618*	*0.720*	*0.548*	*0.742*	*0.761*	*0.462*
*High-alpha*	***0.004***	*0.486*	*0.151*	*0.348*	*0.773*	*0.558*
*Peak-latency*	***0.005***	*0.731*	*0.179*	*0.300*	*0.393*	*0.603*
*Peak-magnitude*	***0.002***	*0.766*	*0.402*	*0.314*	*0.530*	*0.417*
***r value***						
Low-alpha	0.126	0.091	0.152	0.084	0.077	−0.185
High-alpha	**0.644**	0.176	0.353	−0.234	0.073	−0.148
Peak-latency	**0.636**	0.087	0.331	−0.259	−0.214	0.131
Peak-magnitude	**0.676**	0075	0.211	−0.251	−0.158	0.203

*Correlations with *p* value < 0.005 are significant after FDR correction, corresponding to a FDR adjusted *p* value of 0.05. Note: *p* values are in italicized font and significant values are marked in bold.*

Next, we applied FDR correction (Benjamini and Hochberg, 1995) considering the 24 correlations performed. To be statistically significant after such correction, a correlation should present a *p* value < 0.005. As can be observed in Table 1, the three significant correlations (i.e., mean amplitude of high-alpha ERD, latency and magnitude of the alpha ERD peak with the occurrence of the dominant microstate) discussed above remain statistically significant. Notably, because the correlation between the mean amplitude of low alpha and the microstate metrics was not significant, this ERD index was not considered in the following analyses.

Furthermore, to verify the specificity of the above significant correlations for the dominant microstate, we used the Olkin’s test (Marshall and Olkin, 1967) comparing the power of correlations of dominant and non-dominant microstates for dependent correlations. Results reported in Table 2, after FDR correction, show that the correlations between the three alpha ERD indices (i.e., high-alpha mean amplitude, alpha peak latency, and alpha peak magnitude) and the occurrence of the dominant microstate are significantly different (*p* < 0.05) from the correlations between these alpha ERD indices and the occurrence of the non-dominant microstate except for the high-alpha mean amplitude showing a difference between dominant and non-dominant C3 microstate tending toward significance (*p* = 0.052).

**TABLE 2:** *P* values of comparison between correlations with Olkin’s test.

	***DT-C2***	***DT-C3***
High-alpha	**0.033**	0.052
Peak-latency	**0.017**	**0.046**
Peak-magnitude	**0.009**	**0.006**

**P* values ≤ 0.033 indicate a statistically significant difference between dominant and non-dominant microstates after FDR correction, corresponding to an FDR-adjusted *p* value of 0.05. Significant values are marked in bold.*

Finally, we investigated the presence/absence of brain/behavioral correlations for both the three alpha ERD markers and the occurrence of the dominant microstate. Results reported in Table 3 show a negative correlation between reaction time and the mean of high-alpha ERD (*r* = −0.59, *p* = 0.01) and the peak magnitude of the alpha ERD (*r* = −0.64, *p* = 0.005), indicating that subjects with stronger de-synchronization tend to respond more rapidly. On the contrary, a similar correlation was not observed between reaction time and the occurrence of the dominant microstate and the alpha ERD peak latency (*p* > 0.05). Also, for this set of correlations, the FDR correction was applied.

**TABLE 3:** *P* and *r* values of Spearman-rank correlations between microstate and alpha de-synchronization features (occurrence of the dominant pre-stimulus microstate, mean amplitude of high-alpha ERD, and peak magnitude and latency of the alpha ERD) and behavioral performance (accuracy and reaction time).

	***DT-occ***	***High-alpha***	***Peak-latency***	***Peak-magnitude***
***p value***				
Accuracy	0.419	0.672	*0.754*	0.912
Reaction Time	0.253	**0.011**	0.569	**0.005**
***r value***				
Accuracy	−0.203	−0.107	−0.079	+0.028
Reaction Time	−0.284	**−0.593**	−0.144	**−0.641**

*Correlations with *p* values ≤ 0.011 are significant after FDR correction.*

Overall, these results show that only the occurrence of the dominant microstate is linked with the alpha ERD markers.

## Discussion

In the present study, we aimed at investigating the link between the pre-stimulus EEG microstates and other well-known EEG markers during a semantic memory task. To this aim, we investigated the correlations between the features (i.e., the GEV and the FOO) of the dominant and non-dominant pre-stimulus microstates and the anticipatory alpha power reduction that is widely interpreted as a marker of an activated cortical region with increased excitability (Pfurtscheller, 2001). On the contrary, an increment of the alpha power has been proposed as a functional correlate of inhibition in different cognitive tasks (Pfurtscheller, 2001). We observed a positive correlation between the occurrence of the dominant microstate and the three ERD indices (i.e., mean amplitude of the high-alpha ERD and peak magnitude and latency of the alpha ERD). Conversely, no significant correlation was reported between the GEV and any of the anticipatory alpha ERD indices nor between any of the non-dominant microstate features and any of the alpha ERD indices. The present results suggest that only the occurrence of the dominant pre-stimulus microstate has to be considered a valid indicator of the human cortical activation such that a higher number of occurrences in the period preceding a signaled target suggests a stronger cortical activation.

Compared to other EEG correlates that are local and require a period of a certain minimum duration, the EEG microstates have been proposed as a widespread measure of the temporary human brain activity that does not change continuously but remains quasi-stable for epochs of about 80–120 ms (Lehmann et al., 2005). The topography of the EEG microstates has been deeply investigated at rest (Britz et al., 2010; Croce et al., 2018a), and several studies associated such topographies with other EEG correlates and with BOLD activity. Specifically, Britz et al. (2010) reported a link between the BOLD activations in areas belonging specifically to human networks and the four common resting EEG microstate topographies (A, B, C, and D), and more recently, the emergence of these four microstate topographies at rest was linked with intra-cortical alpha oscillations (Milz et al., 2017). Parallel, several other studies have investigated the topographies of the microstates during a variety of tasks (Britz et al., 2008, 2011, 2014; Croce et al., 2018b) showing that the topography that immediately precedes a stimulus influences the post-stimulus event-related potential maps (Lehmann et al., 1994) such that the global temporary human brain state plays a relevant role in information processing (Kondakor et al., 1997). Notably, the A, B, C, and D labeling used at rest was not applied to the pre-stimulus microstates. Although there are increasing numbers of studies investigating the pre-stimulus microstates during the task execution, no direct link with other EEG markers has been yet reported. Importantly, microstate analysis allows us to compute different metrics, such as the GEV and the FOO. In particular, it is commonly accepted that the FOO of a microstate can be thought of as the tendency of its neural generators to become more active. On the other hand, the GEV, indicating the percentage of total variance explained by a specific microstate (Brodbeck et al., 2012), is interpreted to reflect the relative time coverage of its subtended neural generators compared to others (Khanna et al., 2015).

Here, as a first interesting result, we report a positive correlation of the occurrence of the dominant microstate with the mean amplitude of high-alpha ERD but not with that of the low alpha. Such specificity was not investigated in the time-frequency analysis because, in the low-alpha sub-band, a clear ERD peak was not detectable in the majority of subjects, and some of them presented an ERS peak. It was widely suggested that the high-frequency alpha rhythms would reflect task-related oscillations of selective neural systems involved in the processing of task-specific information, whereas the low-frequency alpha rhythms would extensively regulate global human brain arousal and alertness (Klimesch et al., 1998a; Foxe and Snyder, 2011). Interestingly, we previously reported that the topography of the pre-stimulus microstate is related to the current task (Croce et al., 2018b) rather than it having a general functional role for event anticipation and preparation. Specifically, when subjects performed a visuospatial or a semantic memory task, respectively, we reported two distinct pre-stimulus microstate’s topographies. Thus, the specificity of the present correlations with the high-alpha ERD, at least in the stationary analysis, suggests that only the occurrence of the dominant pre-stimulus microstate is strictly linked to the present task rather than a general functional role. Furthermore, a stronger positive correlation between the occurrence of the dominant microstate and the peak magnitude of the alpha ERD was reported. It should be noted that the present paradigm used a fixed cue-target period. Hence, a precise temporal alignment of a preparatory set for the task execution was allowed by the informative cue. Studies using similar paradigms have previously observed that the preparatory alpha ERD tends to increase (in absolute value), rhythmically peaking in proximity to the target onset (Rohenkohl and Nobre, 2011; Spadone et al., 2017). This is important for our study because the dominant pre-stimulus microstate has been located in the latter part of the expectancy period (i.e., 50 ms before the event; Croce et al., 2018b), and the present paradigm allows us to correlate the characteristics of the dominant pre-stimulus microstates with different anticipatory alpha ERD measures, thus providing a more robust result in support of our conclusion. In this regard, the stronger correlation observed with the peak value of alpha ERD rather than its mean value is consistent with the idea that attention can be entrained to the temporal structure of timely stimulus onset, and as observed in the anticipatory alpha ERD, also the dominant pre-stimulus microstate closely tracks the development of temporal expectations. This result is also supported by the positive correlation between the occurrence of the microstate and the alpha ERD peak latency. Because the topography of the microstates provides an overall view of the brain activity in the sub-second range compared to the alpha ERD that requires a period of one or more seconds, the present findings seem to suggest that it can be used to study the dynamics of cortical activation patterns better than other, more local EEG correlates. On the other hand, it can be argued that the significant brain/behavior correlation observed between the alpha ERD indices (i.e., mean and peak amplitude) with the reaction time was lost when the behavioral response was correlated with the occurrence of the dominant microstate, thus suggesting a limitation for this EEG marker. Nevertheless, also for the alpha de-synchronization, such correlation is not consistent across neuroimaging studies. For example, within the attention domain, in two studies of our group requiring the execution of two slightly different visuospatial attention tasks, we reported the presence (Capotosto et al., 2009) or the absence (Capotosto et al., 2017), respectively, of significant correlation between alpha ERD and behavioral results. With this point of view, the lack of relationship between microstates and behavior might not be general but limited to the present experimental task. Future studies might address this issue using a dedicated paradigm involving semantic decisions as well as other cognitive domains. Moreover, these studies should also consider collecting a larger number of data to enhance the proportion of trials per template that might be considered as a limitation of the present study.

Finally, because, in the present study, we reported that the non-dominant microstates also occurred frequently (at least C2 and C3), it could be argued that they should have a functional role related to the present task. At this stage of the research, we cannot speculate on this issue because it is not possible to disentangle results from possible different cognitive processes (i.e., motor, linguistic, attention) present within the current semantic memory task, but future studies, employing a dedicated paradigm in which distinct cognitive processes may be isolated, will assess whether the non-dominant microstate in one task becomes dominant for the other one and vice versa.

## Conclusion

The present study showed a monotonic relationship between the occurrence of the dominant pre-stimulus EEG microstates and the alpha ERD indices during the execution of a semantic memory task, suggesting that only this characteristic of the microstates should be considered as a promising electrophysiological correlate of the cortical activation. This study paves the way to further investigation aiming to correlate the features of the microstates to other EEG markers during different cognitive tasks.

## Data Availability Statement

The dataset generated during the current study is available from the corresponding author on reasonable request.

## Ethics Statement

The studies involving human participants were reviewed and approved by Institutional Review Board and Ethics Committee of the University of Chieti (Prot. No. 1123/2014). The patients/participants provided their written informed consent to participate in this study.

## Author Contributions

PCa recorded and collected EEG data. SS and PCr performed the data analysis. All authors participated in the study design, scientific discussion, and manuscript preparation.

## Conflict of Interest

The authors declare that the research was conducted in the absence of any commercial or financial relationships that could be construed as a potential conflict of interest.

## References

[B1] Babiloni C. Del Percio C. Arendt-Nielsen L. Soricelli A. Romani G. L. Rossini P. M. (2014). Cortical EEG alpha rhythms reflect task-specific somatosensory and motor interactions in humans. *Clin. Neurophysiol. Off. J. Int. Fed. Clin. Neurophysiol.* 125 1936–1945. 10.1016/j.clinph.2014.04.021 24929901

[B2] Benjamini Y. Hochberg Y. (1995). Controlling the false discovery rate: a practical and powerful approach to multiple testing. *J. R. Stat. Soc. Ser. B Methodol.* 57 289–300.

[B3] Bertinetto P. M. Buran C. Laudanna A. Marconi L. Ratti D. Rolando C. (2005). *Corpus e Lessico di Frequenza dell’Italiano Scritto (CoLFIS).* Available online at: http://linguistica.sns.it/CoLFIS/Home.htm

[B4] Britz J. Díaz Hernàndez L. Ro T. Michel C. M. (2014). EEG-microstate dependent emergence of perceptual awareness. *Front. Behav. Neurosci.* 8:163. 10.3389/fnbeh.2014.00163 24860450 PMC4030136

[B5] Britz J. Landis T. Michel C. M. (2008). Right parietal brain activity precedes perceptual alternation of bistable stimuli. *Cereb. Cortex* 19 55–65. 10.1093/cercor/bhn056 18424780

[B6] Britz J. Pitts M. A. Michel C. M. (2011). Right parietal brain activity precedes perceptual alternation during binocular rivalry. *Hum. Brain Mapp.* 32 1432–1442. 10.1002/hbm.21117 20690124 PMC6869880

[B7] Britz J. Van De Ville D. Michel C. M. (2010). BOLD correlates of EEG topography reveal rapid resting-state network dynamics. *Neuroimage* 52 1162–1170. 10.1016/j.neuroimage.2010.02.052 20188188

[B8] Brodbeck V. Kuhn A. von Wegner F. Morzelewski A. Tagliazucchi E. Borisov S. (2012). EEG microstates of wakefulness and NREM sleep. *Neuroimage* 62 2129–2139. 10.1016/j.neuroimage.2012.05.060 22658975

[B9] Capotosto P. Babiloni C. Romani G. L. Corbetta M. (2009). Frontoparietal cortex controls spatial attention through modulation of anticipatory alpha rhythms. *J. Neurosci.* 29 5863–5872. 10.1523/JNEUROSCI.0539-09.2009 19420253 PMC2692025

[B10] Capotosto P. Baldassarre A. Sestieri C. Spadone S. Romani G. L. Corbetta M. (2017). Task and regions specific top-down modulation of alpha rhythms in parietal cortex. *Cereb. Cortex N. Y.* 27 4815–4822. 10.1093/cercor/bhw278 27600845 PMC6433177

[B11] Capotosto P. Spadone S. Tosoni A. Sestieri C. Romani G. L. Della Penna S. (2015). Dynamics of EEG rhythms support distinct visual selection mechanisms in parietal cortex: a simultaneous transcranial magnetic stimulation and EEG study. *J. Neurosci.* 35 721–730. 10.1523/JNEUROSCI.2066-14.2015 25589765 PMC4293418

[B12] Croce P. Zappasodi F. Capotosto P. (2018a). Offline stimulation of human parietal cortex differently affects resting EEG microstates. *Sci. Rep.* 8:1287. 10.1038/s41598-018-19698-z 29352255 PMC5775423

[B13] Croce P. Zappasodi F. Spadone S. Capotosto P. (2018b). Magnetic stimulation selectively affects pre-stimulus EEG microstates. *Neuroimage* 176 239–245. 10.1016/j.neuroimage.2018.04.061 29723638

[B14] Delorme A. Makeig S. (2004). EEGLAB: an open source toolbox for analysis of single-trial EEG dynamics including independent component analysis. *J. Neurosci. Methods* 134 9–21. 10.1016/j.jneumeth.2003.10.009 15102499

[B15] Foxe J. J. Snyder A. C. (2011). The role of alpha-band brain oscillations as a sensory suppression mechanism during selective attention. *Front. Psychol.* 2:154. 10.3389/fpsyg.2011.00154 21779269 PMC3132683

[B16] Jensen O. Gelfand J. Kounios J. Lisman J. E. (2002). Oscillations in the alpha band (9-12 Hz) increase with memory load during retention in a short-term memory task. *Cereb. Cortex N. Y. N* 1991 877–882. 10.1093/cercor/12.8.877 12122036

[B17] Khanna A. Pascual-Leone A. Michel C. M. Farzan F. (2015). Microstates in resting-state EEG: current status and future directions. *Neurosci. Biobehav. Rev.* 49 105–113. 10.1016/j.neubiorev.2014.12.010 25526823 PMC4305485

[B18] Klimesch W. (1999). EEG alpha and theta oscillations reflect cognitive and memory performance: a review and analysis. *Brain Res. Rev.* 29 169–195. 10.1016/S0165-0173(98)00056-3 10209231

[B19] Klimesch W. Doppelmayr M. Russegger H. Pachinger T. Schwaiger J. (1998a). Induced alpha band power changes in the human EEG and attention. *Neurosci. Lett.* 244 73–76. 10.1016/s0304-3940(98)00122-0 9572588

[B20] Klimesch W. Freunberger R. Sauseng P. (2010). Oscillatory mechanisms of process binding in memory. *Neurosci. Biobehav. Rev.* 34 1002–1014. 10.1016/j.neubiorev.2009.10.004 19837109

[B21] Klimesch W. Russegger H. Doppelmayr M. Pachinger T. (1998b). A method for the calculation of induced band power: implications for the significance of brain oscillations. *Electroencephalogr. Clin. Neurophysiol. Potentials Sect.* 108 123–130. 10.1016/S0168-5597(97)00078-6 9566625

[B22] Kondakor I. Lehmann D. Michel C. M. Brandeis D. Kochi K. Koenig. (1997). Prestimulus EEG microstates influence visual event-related potential microstates in field maps with 47 channels. *J. Neural Transm.* 104 161–173. 10.1007/BF01273178 9203079

[B23] Lehmann D. Faber P. L. Galderisi S. Herrmann W. M. Kinoshita T. Koukkou M. (2005). EEG microstate duration and syntax in acute, medication-naive, first-episode schizophrenia: a multi-center study. *Psychiatry Res.* 138 141–156. 10.1016/j.pscychresns.2004.05.007 15766637

[B24] Lehmann D. Michel C. M. Pal I. Pascual-Marqui R. (1994). Event-related potential maps depend on prestimulus brain electric microstate map. *Int. J. Neurosci.* 74 239–248. 10.3109/00207459408987242 7928108

[B25] Marshall A. W. Olkin I. (1967). A multivariate exponential distribution. *J. Am. Stat. Assoc.* 62 30–44. 10.1080/01621459.1967.10482885

[B26] Milz P. Pascual-Marqui R. D. Achermann P. Kochi K. Faber P. L. (2017). The EEG microstate topography is predominantly determined by intracortical sources in the alpha band. *Neuroimage* 162 353–361. 10.1016/j.neuroimage.2017.08.058 28847493

[B27] Min B.-K. Park J. Y. Kim E. J. Kim J. I. Kim J.-J. Park H.-J. (2008). Prestimulus EEG alpha activity reflects temporal expectancy. *Neurosci. Lett.* 438 270–274. 10.1016/j.neulet.2008.04.067 18486342

[B28] Murray M. M. Brunet D. Michel C. M. (2008). Topographic ERP analyses: a step-by-step tutorial review. *Brain Topogr.* 20 249–264. 10.1007/s10548-008-0054-5 18347966

[B29] Oldfield R. C. (1971). The assessment and analysis of handedness: the Edinburgh inventory. *Neuropsychologia* 9 97–113. 10.1016/0028-3932(71)90067-4 5146491

[B30] Pascual-Marqui R. D. Michel C. M. Lehmann D. (1995). Segmentation of brain electrical activity into microstates: model estimation and validation. *IEEE Trans. Biomed. Eng.* 42 658–665. 10.1109/10.391164 7622149

[B31] Pfurtscheller G. (2001). Functional brain imaging based on ERD/ERS. *Vis. Res.* 41 1257–1260. 10.1016/S0042-6989(00)00235-2 11322970

[B32] Rohenkohl G. Nobre A. C. (2011). α oscillations related to anticipatory attention follow temporal expectations. *J. Neurosci. Off. J. Soc. Neurosci.* 31 14076–14084. 10.1523/JNEUROSCI.3387-11.2011 21976492 PMC4235253

[B33] Schwarzkopf D. S. De Haas B. Rees G. (2012). Better ways to improve standards in brain-behavior correlation analysis. *Front. Hum. Neurosci.* 6:200 10.3389/fnhum.2012.00200 PMC3397314 22811662

[B34] Spadone S. Sestieri C. Baldassarre A. Capotosto P. (2017). Temporal dynamics of TMS interference over preparatory alpha activity during semantic decisions. *Sci. Rep.* 7:2372. 10.1038/s41598-017-02616-0 28539601 PMC5443784

[B35] Tallon-Baudry C. Bertrand O. Delpuech C. Permier J. (1997). Oscillatory gamma-band (30-70 Hz) activity induced by a visual search task in humans. *J. Neurosci. Off. J. Soc. Neurosci.* 17 722–734. 10.1523/JNEUROSCI.17-02-00722.1997 8987794 PMC6573221

[B36] Vanni S. Revonsuo A. Hari R. (1997). Modulation of the parieto-occipital alpha rhythm during object detection. *J. Neurosci.* 17 7141–7147. 10.1523/JNEUROSCI.17-18-07141.1997 9278548 PMC6573275

